# Caregiver Resilience as a Protective Factor for Elder Abuse

**DOI:** 10.1093/geroni/igaa057.149

**Published:** 2020-12-16

**Authors:** Elsie Yan

**Affiliations:** The Hong Kong Polytechnic University, Hong Kong, China

## Abstract

This study examines the protective function of caregiving resilience and caregiving self-efficacy for elder abuse. A convenient sample of 600 family caregivers of community dwelling older Chinese in Hong Kong were individually interviewed. Participants were assessed on the caregiving context, care recipient physical functioning and behavioral problems, perceived caregiver stress, neurotic personality, caregiving self-efficacy and resilience. Past year elder abuse was assessed using Revised Conflict Tactic Scale and Older Adult Financial Exploitation. Elder abuse is common in this sample: 7.5, 11.5, 24 per cent reported physical, psychological, and financial abuse respectively. Injuries were reported in 2.3% of the sample. Hierarchical linear regression analysis was conducted. Number of co-residing days and hours of care provided per week were entered into Model One; Care recipient physical functioning, behavioral problems, and caregiver stress were entered into Model Two; Caregiver resilience and caregiving self-efficacy were entered into Model Three. Care recipient behavioral problems and caregiver stress were prominent factors associated with abuse across all models. With the exception of physical abuse, caregiver resilience buffered the effects of care recipient behavioral problems and caregiver stress on all forms of elder abuse. No such effect was observed for caregiving self-efficacy. Intervention efforts aiming at fostering caregiver resilience could potentially prevent elder abuse.

